# Sensitivity and Specificity of Serial Focused Assessment With Sonography in Trauma (FAST) in Patients With Blunt Abdominal Trauma

**DOI:** 10.7759/cureus.92103

**Published:** 2025-09-11

**Authors:** Anirudh Kakran, Rajkumar Singh, Pooja Chaudhary, Ashish K Chaudhary, Mohammad Saquib Ali

**Affiliations:** 1 Department of General Surgery, Ganesh Shankar Vidyarthi Memorial Medical College, Kanpur, IND; 2 Department of Community and Family Medicine, Ganesh Shankar Vidyarthi Memorial Medical College, Kanpur, IND

**Keywords:** blunt abdomen trauma, cect whole abdomen, cect whole abdomen in trauma, focused assessment with sonography in trauma (fast), imaging in trauma, trauma

## Abstract

Introduction

Blunt abdominal trauma (BAT) is a major cause of morbidity and mortality, most frequently resulting from road traffic accidents and falls. Focused assessment with sonography in trauma (FAST) is a rapid, non-invasive, bedside imaging technique used to detect intra-abdominal free fluid. While FAST is widely accessible and radiation-free, its diagnostic accuracy varies with time and clinical context. This study is aimed to evaluate the diagnostic accuracy of serial FAST performed at 4, 8, and 12 hours after injury, using contrast-enhanced computed tomography (CECT) whole abdomen as the reference.

Materials and methods

This was a prospective observational study, conducted at the Department of General Surgery, Ganesh Shankar Vidyarthi Memorial Medical College, Kanpur, Uttar Pradesh, India, between April 2024 and April 2025. A total of 132 adult patients presenting with BAT in emergency were enrolled based on strict inclusion and exclusion criteria. FAST was performed at 4, 8, and 12 hours post-injury. Diagnostic performance was assessed by comparing FAST findings with CECT results in terms of sensitivity, specificity, positive predictive value (PPV), and negative predictive value (NPV).

Results

Liver injuries were the most common (37.12%), followed by splenic and gastrointestinal injuries (18.18% each). Sensitivity of FAST improved from 64.4% at 4 hours to 76.3% at 8 hours and 88.1% at 12 hours. Specificity also increased from 64.3% to 85.7% across the same intervals. PPV remained consistently high (>93%), whereas NPV increased from 17.6% to 46.2%. The diagnostic improvement over time was statistically significant (p < 0.05 for 8 and 12 hours).

Conclusion

Serial FAST enhances diagnostic accuracy in BAT, particularly when performed at delayed intervals. While a positive FAST is highly predictive of injury, a negative FAST, especially at earlier time points, should be interpreted with caution. Integration of serial FAST into trauma protocols can improve injury detection and clinical outcomes.

## Introduction

Blunt abdominal trauma (BAT) is a leading cause of injury-related morbidity and mortality worldwide, most commonly resulting from road traffic accidents, falls, and direct abdominal impacts, including occupational hazards and physical assault [1]. Nearly 85% of abdominal injuries are blunt in nature [2]. If left undiagnosed or improperly managed, patients may develop life-threatening complications such as internal bleeding, sepsis, and multi-organ failure.

Focused assessment with sonography for trauma (FAST) is a rapid, bedside ultrasound technique used to identify free fluid in the peritoneal cavity. Its advantages include wide availability, cost-effectiveness, repeatability, and the absence of radiation exposure [3]. However, limitations such as operator dependence and lower sensitivity reduce its reliability as a standalone diagnostic tool [4].

While FAST is widely utilized in emergency trauma settings, there remains uncertainty regarding its diagnostic accuracy at different time intervals following injury. Injuries with delayed fluid accumulation may initially be missed but could be detected on repeat examinations.

Therefore, the present study was conducted to evaluate the diagnostic accuracy of serial FAST examinations performed at 4, 8, and 12 hours in patients with BAT, using contrast-enhanced computed tomography (CECT) as the reference standard.

## Materials and methods

This prospective study was performed at the Department of General Surgery, Ganesh Shankar Vidyarthi Memorial (GSVM) Medical College, Kanpur, Uttar Pradesh, India, between April 2024 and April 2025. The patients who presented to our hospital within this time frame with a history of blunt trauma to the abdomen and age above 18 years were considered for the study. The study excluded patients with penetrating trauma to the abdomen, associated firearm injury, pre-existing malignant condition, history of chronic liver disease, portal hypertension, chronic kidney disease, congestive heart failure, allergic reaction to contrast, and ascites due to any cause. Patients requiring emergency laparotomy and hemodynamically unstable patients were excluded.

The study used systematic sampling. The study enrolled 132 patients who fulfilled inclusion and exclusion criteria. For every patient, appropriate informed consent was acquired. The study adhered to ethical guidelines, with approval from the Ethics Committee of GSVM Medical College (approval number: EC/160/April/2024; reference number: EC/BMHR/2024/81; dated: 18-01-2024).

Patients were kept under observation, and vitals were monitored regularly. FAST examination was done at 4 hours, 8 hours, and 12 hours. Meanwhile, CECT whole abdomen of the patient was also performed. Patients who had to undergo emergency surgical intervention during the time of observation were not included in this study.

FAST relies on real-time B-mode imaging and is performed with the patient in a supine position at the examiner’s waist level. The scanning process usually begins on the patient’s right side. For proper orientation, the probe indicator should face the patient’s right side during transverse imaging, and it should point toward the head for sagittal views.

The right upper quadrant is the first region commonly assessed. The goal is to visualize Morrison’s pouch, which is the potential space between the liver and the right kidney. The curvilinear probe is placed along the mid-to-posterior axillary line, typically between the 8th and 11th intercostal spaces. The probe is gently swept anteriorly and posteriorly to capture a thorough view of this area. To expand the field of evaluation, the probe is moved downward to assess the liver tip and right paracolic gutter and then angled upward to visualize the diaphragm and lower thoracic cavity, utilizing the liver as an acoustic window.

Evaluation of the pericardium is performed by placing the probe just below the xiphoid process in a transverse orientation. Depth should be adjusted to ensure that the entire pericardium and cardiac silhouette are clearly visible, again using the liver as an acoustic window. A scanning angle of less than 15 degrees is optimal for this subxiphoid approach, and an overhand grip on the probe can help maintain this shallow angle. If visualization proves difficult, the patient may be instructed to take a deep breath to reposition the heart.

The left upper quadrant (LUQ) examination targets the peri-splenic and splenorenal recesses. This view is more challenging due to the spleen’s smaller size compared to the liver and because the dependent spaces in the LUQ are more posterior and cephalad. The probe is positioned sagittally along the posterior axillary line, between the sixth and ninth ribs. The operator should fan the probe anteriorly and posteriorly to assess the peri-splenic and splenorenal areas. A cephalad sweep allows visualization of the subphrenic space and lower thoracic cavity, while a caudal sweep helps evaluate the left paracolic gutter.

The suprapubic region is examined by placing the probe just above the pubic symphysis in a transverse orientation. A full bladder enhances the sonographic window; thus, if the bladder is decompressed, angling the probe caudally may help. Increasing the scanning depth allows evaluation of the posterior bladder wall, where free fluid may accumulate. The operator should sweep the probe both caudally and cranially to inspect the most dependent spaces of the pelvis: the rectovesical space in males, and the rectouterine (pouch of Douglas) and vesicouterine pouches in females. For enhanced visualization in female patients, the probe may be rotated 90 degrees clockwise into a sagittal orientation, offering a clearer view of these pelvic recesses.

SPSS Version 26.0 (IBM Corp., Armonk, NY, USA) was used to analyze data. Categorical variables (e.g., gender, organ injured) were expressed as frequencies as well as percentages. Fisher's exact or chi-square test for categorical variables was employed to compare groups. P-values of <0.05 were considered statistically significant.

## Results

In this study group, as shown in Table 1, liver injury emerged as the most common type, occurring in 49 cases, which represents 37.12% of the total patients. Splenic injury and gastrointestinal injury were each observed in 24 cases, accounting for 18.18%, respectively. Mesenteric injury was reported in 14 cases, which represents 10.61% of the total. Urinary system injury was relatively uncommon, documented in only five cases, comprising 3.79% of the overall study population.

**Table 1:** Distribution of injuries as per CECT whole abdomen CECT, contrast-enhanced computed tomography

Injury Type	Count	Percentage (%)
Liver injury	49	37.12%
Splenic injury	24	18.18%
Gastrointestinal injury	24	18.18%
Urinary system injury	5	3.79%
Mesenteric injury	14	10.61%

As shown in Table 2, FAST examination performed at 4 hours in detecting injuries showed that out of the total cases, FAST correctly identified the presence of injury in 76 (57.6%) patients, classified as true positives. In five (3.8%) cases, the test incorrectly indicated an injury when none was present, representing false positives. Conversely, FAST failed to detect existing injuries in 42 (31.8%) cases, categorized as false negatives. In nine (6.8%) cases, FAST accurately ruled out the presence of injury, corresponding to true negatives.

**Table 2:** FAST findings at 4 hours FAST, focused assessment with sonography in trauma

FAST at 4 Hours	Categorization of Result for Injury Present	Counts	% of Total
Positive	True positive	76	57.6%
	False positive	5	3.8%
Negative	False negative	42	31.8%
	True negative	9	6.8%

As shown in Table 3, FAST at 8 hours correctly identified injuries in 90 (68.2%) cases, which are classified as true positives. In three (2.3%) cases, FAST indicated the presence of an injury where none existed, classified as false positive. FAST failed to detect actual injuries in 28 (21.2%) cases, classified as false negatives. FAST accurately ruled out injuries in 11 (8.3%) cases, classified as true negatives.

**Table 3:** FAST findings at 8 hours FAST, focused assessment with sonography in trauma

FAST at 8 Hours	Categorization of Result for Injury Present	Counts	% of Total
Positive	True positive	90	68.2%
	False positive	3	2.3%
Negative	False negative	28	21.2%
	True negative	11	8.3%

As shown in Table 4, FAST at 12 hours successfully identified injuries in 104 (78.8%) cases, categorized as true positives. FAST identified injury where none existed in two (1.5%) cases, classified as false positives. FAST failed to detect existing injuries in 14 (10.6%) cases, classified as false negatives. FAST accurately ruled out the presence of injury in 12 (9.1%) cases, categorized as true negatives.

**Table 4:** FAST findings at 12 hours FAST, focused assessment with sonography in trauma

FAST at 12 Hours	Categorization of Result for Injury Present	Counts	% of Total
Positive	True positive	104	78.8%
	False positive	2	1.5%
Negative	False negative	14	10.6%
	True negative	12	9.1%

As shown in Table 5, diagnostic accuracy of the FAST examination improves progressively over time. Sensitivity increases from 64.4% at 4 hours to 76.3% at 8 hours and further to 88.1% by 12 hours. This indicates that FAST becomes significantly more effective in identifying true injuries as more time elapses after trauma. Specificity also improves with time, which starts at 64.3% at 4 hours, increases to 78.6% at 8 hours, and reaches 85.7% at 12 hours. The positive predictive value (PPV) of FAST remains high throughout, indicating strong reliability when the test result is positive. At 4 hours, the PPV is 93.8%, rising to 96.8% at 8 hours and reaching 98.1% by 12 hours. In contrast, the negative predictive value (NPV) is relatively low in the early hours, making negative results less trustworthy. At 4 hours, the NPV is only 17.6%, improving to 28.2% at 8 hours and 46.2% by 12 hours. Although the NPV increases over time, it remains lower than other metrics, indicating that a negative FAST result becomes more helpful with time but is still not sufficiently reliable for confidently excluding injury on its own.

**Table 5:** Sensitivity, Specificity, PPV, and NPV of FAST at 4 hours, 8 hours, and 12 hours FAST, focused assessment with sonography in trauma; PPV, positive predictive value; NPV, negative predictive value

FAST Time	Sensitivity	Specificity	PPV	NPV	p-Value
4 hours	64.4%	64.3%	93.8%	17.6%	0.0728
8 hours	76.3%	78.6%	96.8%	28.2%	<0.05
12 hours	88.1%	85.7%	98.1%	46.2%	<0.05

## Discussion

The injury distribution in our study showed that liver injuries were the most common (37.1%), followed by splenic and gastrointestinal injuries (18.2% each), mesenteric injuries (10.6%), and urinary system injuries (3.8%). This closely parallels patterns described in previous BAT reports, where solid organs, particularly the liver and spleen, predominate. Bhatia et al. reported a similar distribution in a retrospective analysis of 1,519 adult BAT patients, with liver injury observed in 38.8% and splenic injury in 31.2% [5]. Other large trauma-center series have documented liver injury rates of 36-38% and splenic injury rates of 19-32%, further supporting these findings [6,7]. The predominance of solid organ injury is generally attributed to their size, fixed anatomical position, and rich vascularity, which increase susceptibility to deceleration and compression forces [8]. In contrast, urinary system injuries were least frequent in our series (3.8%), consistent with prior literature indicating that genitourinary trauma represents <10% of intra-abdominal injuries in BAT, often requiring high-energy impact or pelvic fracture [9].

Our study also assessed the diagnostic accuracy of FAST at multiple intervals, demonstrating a progressive increase in sensitivity with serial examinations: 64.4% at 4 hours, 76.3% at 8 hours, and 88.1% at 12 hours. Specificity remained consistently high, and false-positive rates were low throughout. These trends align with meta-analyses reporting moderate overall sensitivity (74-79%) and high specificity (96-99%) for FAST in adults [10,11]. Previous studies, including those by Mattison et al. and McKenney et al., have shown that repeat FAST examinations increase detection rates by revealing delayed free fluid accumulation, particularly in cases with initially low fluid volume or evolving injuries [12,13]. This mirrors our observed improvement over time. Although the NPV improved from 17.6% at 4 hours to 46.2% at 12 hours, it remained suboptimal, echoing prior literature emphasizing that FAST is more reliable for ruling in rather than ruling out intra-abdominal injury [14]. Current recommendations advocate that patients with negative FAST findings but persistent abdominal tenderness, hemodynamic instability, or high-risk mechanisms should undergo further evaluation with CECT or serial FAST to avoid missed hollow-viscus or mesenteric injuries [15,16].

Our study has certain limitations that should be acknowledged. First, it was conducted at a single tertiary care center, which may limit the generalizability of the findings to other settings. Second, the accuracy of the test is inherently operator-dependent, and inter-observer variability could not be completely eliminated. Third, the relatively small sample size may limit the precision of sensitivity and specificity estimates. Fourth, only adult patients were included, which precludes extrapolation of results to pediatric trauma populations where injury patterns and sonographic interpretation may differ. Fifth, patients who were hemodynamically unstable and required immediate surgical intervention could not undergo the planned serial FAST examinations, potentially introducing selection bias.

## Conclusions

Serial FAST is a valuable diagnostic tool in the assessment of BAT, with its sensitivity, specificity, and overall diagnostic accuracy improving significantly over time. While a single FAST examination at 4 hours may yield false-negative results and cannot reliably exclude intra-abdominal injuries, repeat evaluations at 8 and especially 12 hours enhance diagnostic confidence. A positive FAST result is highly predictive of true injury at all time points. However, due to the relatively low NPV, particularly in early assessments, FAST should be complemented by CECT imaging in cases with negative or inconclusive findings. Thus, incorporating serial FAST into trauma protocols can improve early detection, guide timely management, and potentially reduce morbidity and mortality associated with missed injuries.
